# Formation of H_2 _and CH_4 _by weathering of olivine at temperatures between 30 and 70°C

**DOI:** 10.1186/1467-4866-12-6

**Published:** 2011-06-27

**Authors:** Anna Neubeck, Nguyen Thanh Duc, David Bastviken, Patrick Crill, Nils G Holm

**Affiliations:** 1 Department of Geological Sciences, Stockholm University, Sweden; 2 Department of Thematic Studies-Water and Environmental Studies, Linköping University, Sweden

## Abstract

Hydrocarbons such as CH_4 _are known to be formed through the Fischer-Tropsch or Sabatier type reactions in hydrothermal systems usually at temperatures above 100°C. Weathering of olivine is sometimes suggested to account for abiotic formation of CH_4 _through its redox lowering and water splitting properties. Knowledge about the CH_4 _and H_2 _formation processes at low temperatures is important for the research about the origin and cause of early Earth and Martian CH_4 _and for CO_2 _sequestration. We have conducted a series of low temperature, long-term weathering experiments in which we have tested the CH_4 _and H_2 _formation potential of forsteritic olivine.

The results show low temperature CH_4 _production that is probably influenced by chromite and magnetite as catalysts. Extensive analyses of a potential CH_4 _source trapped in the crystal structure of the olivine showed no signs of incorporated CH_4_. Also, the available sources of organic carbon were not enough to support the total amount of CH_4 _detected in our experiments. There was also a linear relationship between silica release into solution and the net CH_4 _accumulation into the incubation bottle headspaces suggesting that CH_4 _formation under these conditions could be a qualitative indicator of olivine dissolution.

It is likely that minerals such as magnetite, chromite and other metal-rich minerals found on the olivine surface catalyze the formation of CH_4_, because of the low temperature of the system. This may expand the range of environments plausible for abiotic CH_4 _formation both on Earth and on other terrestrial bodies.

## Background

The CH_4 _detected in the Martian atmosphere [1-3] in 2004 raised the question whether or not the CH_4 _were formed biotically or abiotically. It was suggested by Krasnopolsky et al. [3] that microorganisms on Mars may have produced it. However, several abiotic processes may be responsible for the detected atmospheric CH_4_, such as volcanism, exogenous sources and serpentinization of ultramafic rocks [4-6]. There are too few hot spots present on Mars to account for the CH_4 _concentrations that were detected and volcanism is not likely to be the major source of CH_4 _on Mars. Neither are the exogenous sources, such as meteorites and comets, for the same reason. Oze and Sharma [4] have calculated reaction rates for olivine dissolution on Mars, using olivine chemical compositions found in the Martian Schergottite-Nakhlite-Chassigny (SNC) meteorites, a temperature of 25°C and varying pH. They came to the conclusion that dissolution of olivine is favorable in the subsurface of Mars at such low temperatures, both kinetically and thermodynamically, which means that serpentinization would be a potential source for CH_4 _detected on the Martian atmosphere.

On the contemporary Earth, there are also CH_4 _seeps and plumes that are suggested to be of abiotic origin, at least to some extent [7-9]. Abiotically formed CH_4 _may provide carbon and energy for microorganisms in the deep subsurface biosphere and may serve as a precursor for forming longer hydrocarbons such as natural gas and oil. This process may be important for CO_2 _sequestration. Basaltic (45-52% SiO_2_) and ultramafic (<45% SiO_2_) hydrothermal systems as well as continental groundwaters host a vast number of bacterial and archaeal organisms [10,11] found at depths down to at least 800 meters below the seafloor (mbsf) [12] and in volcanic glass at depths down to 954 mbsf [13]. Microbial communities are also found in volcanic hot springs, in saline groundwaters at depths exceeding 2 km in igneous rocks, and in continental flood basalts [11]. Some microorganisms living in these environments are chemolithoautothrophs, i.e., they are autotrophic organisms that derive their energy from inorganic compounds such as H_2 _and CH_4 _emanating from rock-associated fluids and gases. An important question is to what extent microorganisms can use the chemical energy released exclusively from the alteration of olivine, one of the most common mineral in the Earth mantle [14-19]. This question bears upon the dynamics of contemporary subsurface microbial communities and the possibilities for such extreme environments to be modern analogues to early Earth ecosystems.

Weathering of olivine is sometimes called serpentinization due to the formation of serpentine minerals as alteration products. Fluids associated with serpentinization hydrothermal vent systems such as Lost City in the Atlantic Ocean often show elevated concentrations of CH_4 _[7], which can be a product of H_2 _reacting with CO_2 _or CO, gases that can be found in hydrothermal systems. Hence the abiotic interaction between water and mafic minerals can result in formation of H_2 _and CH_4 _which both represent high quality electron donors for chemosynthetic organisms (e.g. hydrogenotrophic and methanotrophic microorganisms).

The release of H_2 _from weathering of mafic minerals may be due to either formation through water reduction or release from the mineral itself. Freund et al. [20] suggest that nominally anhydrous minerals such as olivine, contains a considerable amount of H_2 _within its crystal structure in the form of hydroxyl anions (OH^-^) or peroxy links released upon fracturing or heat. The formation of molecular H_2 _may also be coupled to the formation of magnetite (Eq. 1). In that reaction, ferrous iron is oxidized to ferric iron together with the reduction of water to H_2_. However, if the silica activity is high, serpentine or brucite will incorporate the iron into the crystal structure and thus prevent it from becoming oxidized [21] and thus prevent H_2 _formation. The Fischer-Tropsch (FT) reaction (Eq. 2) is widely known in the oil and petroleum industry as an abiotic, catalyzed reaction capable of producing CH_4 _and longer hydrocarbons such as petroleum, waxes and oils [22] from gaseous H_2 _and CO. The usual catalysts for that reaction are magnetite, Co and Ru oxides. The specific formation of CH_4 _from H_2 _and CO_2 _is also called the Fischer-Tropsch Type (FTT) or Sabatier reaction (Eq. 3). The FTT reactions are modified from the FT reaction in the way that the carbon source is CO_2 _instead of CO and the presence of water [23]. This reaction is often used to explain the presence of abiotic CH_4 _and other hydrocarbons in some natural systems on Earth [8]. The formation of CH_4 _in ultramafic natural systems is often thought to be the combination of the FTT reaction linked to the formation of H_2 _through the olivine hydration process [7,24].(1)(2)(3)

FTT reactions are considered to be common in hydrothermal systems and ultramafic rocks and have also been the focus for research considering the abiotic formation of precursors of biologically critical molecules such as amino acids and lipids [7,8,17,25].

Berndt et al. [26] conducted olivine dissolution experiments based on the study of Janecky and Seyfried [27]. They wanted to explicitly study the CH_4 _forming processes coupled to olivine dissolution and serpentinization at 300°C and 500 bars. They could see a distinct increase in CH_4 _throughout the experiments and also an increase in other hydrocarbons such as C_2_H_6 _and C_3_H_8_. The catalyst present in their experiment was exclusively magnetite. Later, Horita et al. [28] confirmed the formation of CH_4 _through serpentinization, but also showed that magnetite is not the only and most efficient catalyst to form CH_4 _in an olivine dissolution environment. Instead, the presence of awaruite (Ni_3_Fe) increased the rate of formation severalfold. Since awaruite is a common associated mineral in ultramafic rocks [29], this approach was highly relevant. Another experiment made by McCollom et al. [30] with the purpose of investigating the formation of hydrocarbons through serpentinization of olivines and with no additional catalysts, showed continuous increase of CH_4 _throughout the experiment. The experiments were conducted under a pressure of 350 bars and 300°C. However, most of the CH_4 _(about 80%) found in these experiments was most likely not formed but was suggested to be released from fluid inclusions and carbon species within the olivine crystals. Another interesting observation in their experiments, though, was the need of fresh mineral surfaces in order to form CH_4 _which was probably due to partial oxidation of the surface. Instead of Ni-bearing catalysts, Foustoukos and Seyfried [23] used a mixture of Cr and Fe oxides (chromite, FeCr_2_O_4_) in an effort to produce hydrocarbons under hydrothermal conditions (390°C and 400 bars). Chromite is commonly associated with olivine-rich rocks and would therefore be part of a natural ultramafic hydrothermal system. The found CH_4 _concentrations were higher than earlier experimental efforts without the presence of Cr,Fe-bearing catalysts. It is now widely accepted that CH_4 _may be produced abiotically though serpentinization reactions at temperatures around 300°C. Previous studies regarding the FTT or Sabatier reactions often considered temperatures over 100°C. High temperatures promote faster reaction rates and lower kinetic barriers but are not suitable for living cells and can only support chemosynthetic life at a distance along a diffusion and temperature gradient within a hydrothermal environment, such as the porous lava layers or the diffuse vents in which hot hydrothermal water is mixed and quenched by downwelling seawater. However, if significant H_2 _formation with additional, indirect formation of reduced compounds occurs at lower temperatures, it would drastically expand the potential environments where such reactions can support microbial life. Studies of olivine alteration at lower temperatures are essential regardless of slower reaction rates and the need of long-term studies. In this study we focus on serpentinization in the temperature range of 30 to 70°C and whether significant formation of H_2 _and CH_4 _can be measured at such temperatures.

## Methods

### Experimental

Olivine sand was incubated with buffered and non-buffered Milli-Q water in glass infusion bottles at three different temperatures: 30, 50 and 70°C. Natural olivine sand (Forsterite 91, Fo91) from North Cape Minerals in Åheim, Norway was used in the dissolution experiments. Grain sizes ranged between 0.125 and 1.00 mm with the majority of the grains between 0.250 and 0.500 mm. We made specific surface area measurements using the B.E.T. method on a Micromeritics ASAP2020 Surface Area and Porosity Analyzer with N_2 _as a carrier gas. Prior to the B.E.T. measurements, ~ 8 g of olivine was degassed at 300°C for 600 min in order to remove any adsorbed gases or liquids.

The olivine sand was washed with deionized water about 15 times until the water around the grains was clear. Thereafter, the material was washed in acetone in an ultrasonic bath for 5 minutes in order to remove finer particles and adsorbed organic material. The sand was dried overnight at 30°C. The 120 mL glass incubation bottles were all washed in deionized water and combusted at 550°C to remove organic carbon compounds.

Approximately 25 g of olivine was weighed and put into each incubation bottle together with 60 mL of liquid. Two different liquids were used in the experiment, 2.2 mM bicarbonate buffer and pure Milli-Q water (with a water resistivity of 18.2 MΩcm) in order to trace the differences in CH_4 _formation with and without added dissolved CO_2_. The pH was measured in all bottles to be between 8.35 and 9.44 and with average values of 9.07 for the pure Milli-Q water and 8.67 for the buffered Milli-Q water. Bottles were sealed with massive 10 mm thick butyl rubber stoppers (Apodan, Denmark) and an alumina crimp seal cap. Blank samples without olivine sand were prepared. To obtain an O_2 _free environment, all bottles were evacuated and flushed with CH_4 _free N_2 _repeatedly three times to an overpressure of 2 bars. The bottles were equilibrated to atmospheric pressure before autoclaving at 140°C for 20 minutes. After sterilization, initial samples were taken and then the bottles were incubated for 9 months at three different incubation temperatures; 30°C, 50°C and 70°C. The bottles were not shaken or stirred.

Conversion between ppm and moles were calculated with the ideal gas law.

## Analysis

### XRD, Microscopy, ESEM

X-ray Diffraction (XRD) was used to identify mineral phases other than olivine in the sand. The analyses were made at the Swedish Geological Survey on a Siemens D5000 theta/theta diffractometer with CuK radiation and a graphite monochromator at 40 kV and 40 mA.

Doubly polished olivine thin sections with a thickness of about 200 μm [31] were analyzed microscopically to identify mineral phases, analyze mineral contacts, and to evaluate the occurrence of fluid inclusions and microstructures. An XL30 environmental scanning electron microscope with a field emission gun (XL30 ESEM-FEG) was used to analyze the mineral surfaces before and after incubation in order to identify possible mineral coating, pitting, etching or other changes to the mineral surface. The ESEM was equipped with an Oxford x-act energy dispersive spectrometer (EDS), backscatter electron detector (BSE) and a secondary electron detector (SE). Peak and element analyses were made using INCA Suite 4.11 software.

### CH_4, _H_2_, Carbon species

Headspace CH_4 _measurements were made on five occasions by injecting 2 mL of N_2 _into the incubation bottles followed by removing 2 mL of gas sample with a syringe which were loaded on a 500 μL injection loop then injected into a gas chromatograph with flame ionization detection (GC-FID, Shimadzu 8A). CH_4 _was separated from the matrix gas with a 2 m × 1/8 " o.d. stainless steel column packed with HayeSep Q 80/100 at 50°C using N_2 _carrier gas. A 99.9 ± 2 ppmv CH_4 _standard was used for calibration (Air Liquide).

Similarly, a 500 μL loop of headspace gas was injected into a gas chromatographer with a reducing compound photometer (Peak Performer Reduced Gas Analyzer PP1) [32] to measure H_2 _and CO. After being separated from the matrix gas through a system of two-packed columns using N_2 _carrier gas, H_2 _was indirectly detected and quantified by the liberated mercury vapor from the heated bed of mercury oxide with a UV (254 nm) absorption photometer. A 10 ± 2 ppmv H_2 _standard (Air Liquide) was used for calibration. CO measurements were made without quantification.

Carbon species were identified and quantified by XPS (X-ray Photoelectron Spectroscopy).

XPS spectra were recorded with a Kratos Axis Ultra DLD electron spectrometer using a monochromated Al Kα source operated at 150 W, a hybrid lens system with magnetic lens providing an analysis area of 0.3 mm × 0.7 mm, and a charge neutralizer. The binding energy (BE) scale was referenced to the C1s line of aliphatic carbon, set at 285.0 eV. Processing of the spectra was accomplished with the Kratos software.

### Trace Elements

To investigate mineral dissolution, liquid phase elemental analyses with Inductively Coupled Plasma Optical Emission Spectroscopy (ICP-OES Spectro, Varian Vista AX) with Ar as a carrier gas and an analytical error of about 4% were made in a subset of the bottles (2 measurements per variable). Samples were prepared by mixing 4 mL of sample solution with 40 μL of HNO_3 _in order to keep the trace metals dissolved. The relative error was ~ 4%.

## Results

### XRD, Microscopy, ESEM

**Figure 1:**
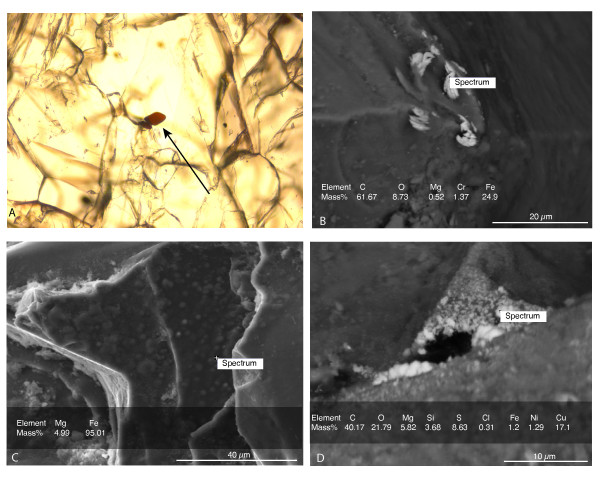
**ESEM spot analyses of potential catalyst sites on the olivine surfaces used in the incubation experiments**. Relative proportions of elements in weight percent is presented in each picture. The high carbon content in picture B and D is due to carbon coating of the sample. A) SEM image of a chrome-spinel crystal taken using optical microscopy, B) SEM image of a Cr and Fe-rich phase C) SEM image of a Fe-rich phase and D) SEM image of Fe, Ni and Cu-rich phases.

**Figure 2:**
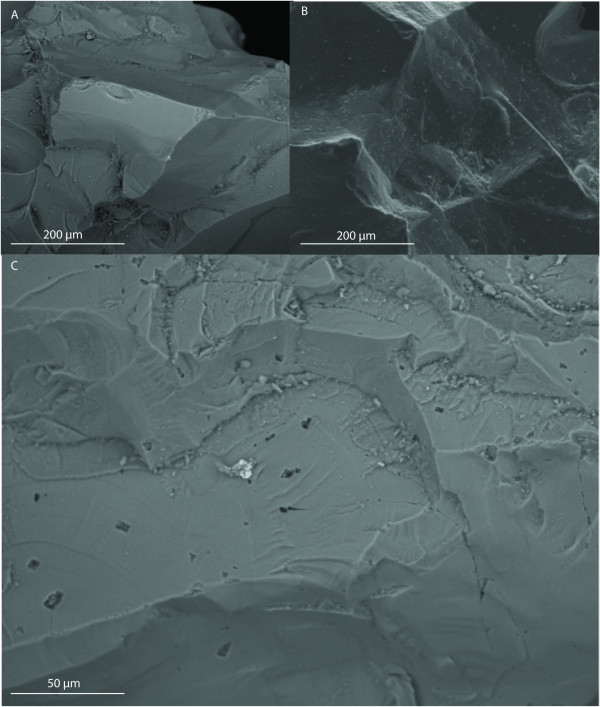
**ESEM pictures showing water influence on olivine surfaces before and after incubation**. An initial olivine grain surface (A) is relatively "clean" from pit marks, etching and precipitates compared with the incubated grains which show a slight increase in small crystal precipitates on the surface (B) and pit marks (C).

The XRD data indicate that the initial forsterite-dominated olivine contained accessory minerals including chlorite, talc, phlogopite and a Fe-Cr-oxide, probably a chromite-magnetite or chrome-bearing chlorite, and small peaks indicative of possible pyroxenes and magnesite, a magnesium carbonate. Chopra et al. [33] define the composition of the Åheim forsteritic olivine as 96% olivine, 4% accessory minerals (mainly pyroxene, clinochlore and phlogopite) and less than 1% spinel. ESEM analyses also show clear Cr, Ni, Cu and Fe-rich areas at localized spots on the grain surface (Figure 1). Microscopic analyses of the initial olivine show both interstitial spinel crystals and well-defined chlorites. Some indications of pitting and precipitations after incubation could be seen with ESEM, see Figure 2. Otherwise, no major changes could be seen with optical microscope.

### CH_4_, H_2_, Carbon species

**Figure 3:**
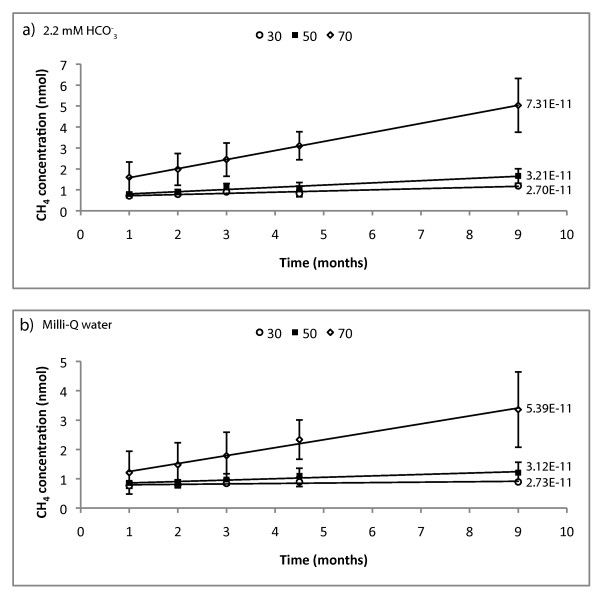
**Accumulation of CH_4 _(in nmol) in the headspace of the incubation bottles as a function of time**. a) is the concentration of CH_4 _in the bottles containing 2.2 mM bicarbonate buffer and the accumulation rates in mol/m^2^/s and b) is the concentration of CH_4 _in the bottles containing only Milli-Q water and the accumulation rates in mol/m^2^/s. All values are after subtracted control values.

After flushing the bottles with CH_4_-free N_2 _and autoclaving, the headspace gas concentration was measured in order to get initial point analyses. Initial gas concentrations in the headspace of the bottles of H_2 _were 0.019 nmol and CH_4 _less than 0.31 nmol, Table 1. CH_4 _accumulated linearly over time in all incubation experiment bottles, (Figures 3a-b). The error bars are calculated from the standard deviation of the concentrations and indicate a larger element of uncertainty in the non-buffered analyses. However, analysis of variance (ANOVA) comparisons showed no substantial differences between buffered and non-buffered CH_4 _formation concentrations (p = 0.42, F = 0.71). There are some clear ANOVA distinctions between the different temperatures (p < 0.0005, F = 41.97).

**Table 1:** The concentrations of CH_4_, H_2_, CO and CO_2 _measured in the experiments

Substance	Temp (°C)	Buffered solution (nmol)	Water solution (nmol)
CH_4 _initial	-	< 0.31	< 0.31
CH_4 _final	30	1.19 ± 0.018	0.89 ± 0.27
CH_4 _final	50	1.66 ± 0.26	1.21 ± 0.33
CH_4 _final	70	5.04 ± 1.18	3.36 ± 1.14
H_2 _initial	-	0.019 ± 0.8E-3	0.019 ± 0.8E-3
H_2 _final	30	5.93 ± 2.09	3.81 ± 0.35
H_2 _final	50	4.71 ± 0.19	3.61 ± 0.79
H_2 _final	70	5.08 ± 0.02	4.46 ± 1.41
CO final	30,50,70	detected	detected
CO_2 _final	30	201.60 ± 44.37	68.84 ±13.54
CO_2 _final	50	484.91 ± 179.56	118.76 ± 46.03
CO_2 _final	70	623.62 ± 122.56	319.25 ± 130.83

No quantification of the CO data was made and the CO_2 _was measured only in the end of the experiments. Both CH_4 _and CO_2 _is temperature dependent whereas H_2 _is not.

**Figure 4:**
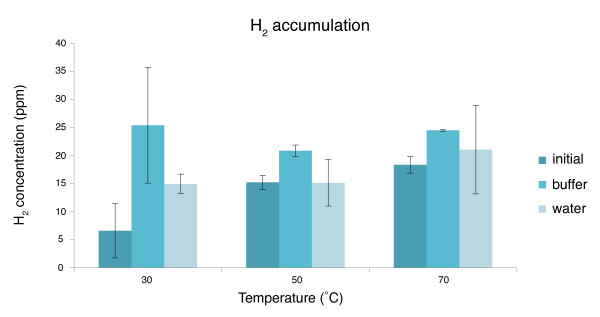
**Concentration of gaseous H_2 _in the headspace of buffered and Milli-Q water incubation bottles**. There is a higher concentration of H_2 _in bottles with 2.2 mM bicarbonate buffer compared with the Milli-Q water bottles. There is no clear temperature dependence on the accumulated concentrations.

The dissolution rates in Table 2 were obtained from the linear regression of the data, Figure 3, and the B.E.T. surface area analyses. The rates of CH_4 _are calculated from the net accumulation in which the controls are subtracted from the samples. pH measurements were made before and after the experiments. The increase in pH in the water samples after 9 months is around 1.31 and in the buffered samples about 0.87 pH units. Final H_2 _concentrations were measured after termination of the experiment, Figure 4.

**Table 2:** Rates of olivine dissolution and CH_4 _accumulation

pH	Initial fluid composition	Grain size (m)	Temp (°C)	Duration of dissolution (days)	Dissolution rate based on Si (Mg) (mol/m^2^/s)	Accumulation rate of CH_4_ (mol/m^2^/s)
8.72	2.2 mM HCO^-^_3_	2.5E-4 to 1E-4	30	295	8.10E-12 (7.28E-12)	2.70E-11
8.74	2.2 mM HCO^-^_3_	2.5E-4 to 1E-4	50	295	1.81E-11 (4.68E-12)	3.21E-11
8.57	2.2 mM HCO^-^_3_	2.5E-4 to 1E-4	70	295	5.12E-11 (1.91E-12)	7.31E-11
9.05	H_2_O (Milli-Q)	2.5E-4 to 1E-4	30	295	6.35E-12 (5.46E-12)	2.73E-11
9.01	H_2_O (Milli-Q)	2.5E-4 to 1E-4	50	295	1.46E-11 (7.41E-12)	3.12E-11
9.09	H_2_O (Milli-Q)	2.5E-4 to 1E-4	70	295	6.39E-11 (1.79E-12)	5.39E-11

Rates of dissolution calculated from the release of Si and Mg into solution and from the accumulation of CH_4 _into the headspace of the experiment bottles after 295 days of dissolution. The CH_4 _rate is calculated from the net accumulation of CH_4 _into the headspace of the bottles, i.e. with the control values subtracted from the analyzed values. The pH is changing between the buffered and non-buffered groups but not remarkably within the groups.

### Trace elements

**Figure 5:**
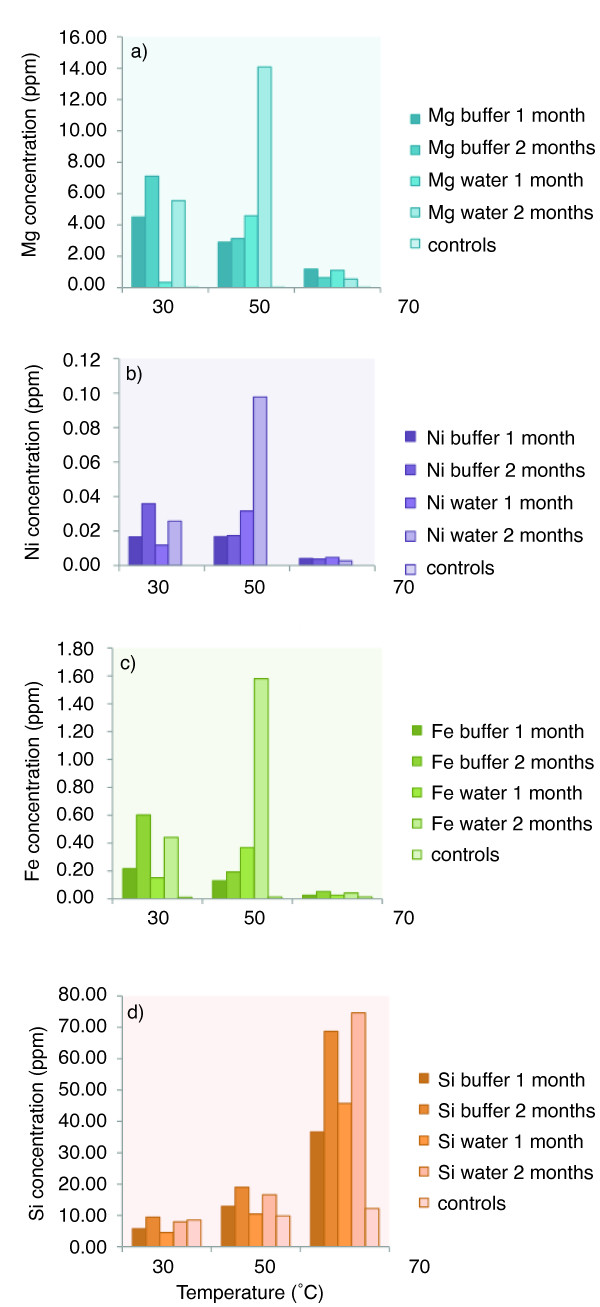
**ICP analyses of elements in solution**. Concentration of elements in ppm released into solution after one and two months of incubation as a function of temperature. Mg (a), Ni (b), Fe (c) and Si (d).

Mg, Ni and Fe concentrations in solution are shown to be temperature and pH dependent (Figure 5 a, b and 5c). In the buffered solutions with stable pH values, there is a decreasing trend of dissolved Mg, Ni and Fe ions. In the non-buffered solutions there is a strong increase in dissolved ions with temperature and time at temperatures below 70°C. In the 70°C treatment the concentrations decreases drastically.

**Figure 6:**
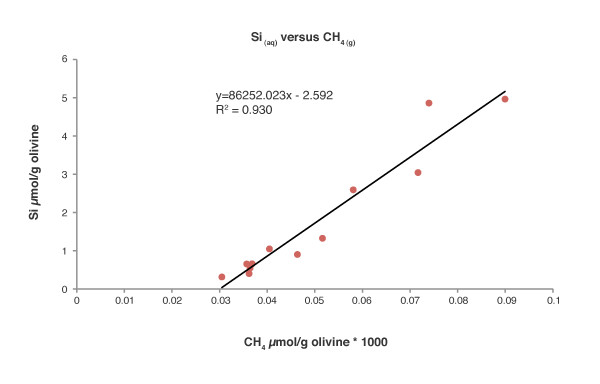
Si release into solution (μmol/g olivine) and formed CH_4 _gas (μmol/g olivine*1000) in the incubation bottle headspace. Random samples of Si from all sample groups were plotted against CH_4_.

An increase of Si over time was observed in all samples (Figure 5d). There is a consistent trend of increasing Si concentration in solution with both temperatures in the buffered and unbuffered samples. There is a linear relationship between the release of Si into solution and CH_4 _accumulation in the headspace (Figure 6). The degree of correlation between the data represented in Figure 6 is very close to 1 (R^2 ^= 0.93) suggesting a strong correlation between the CH_4 _and Si data. The average amount of Si of the olivine sand in the bottles is about 5 g/25 g of olivine. The average wt% loss of Si from the olivine into solution after incubation at 70°C is around 0.035 wt%/month of the initial Si weight and at 30°C the average wt% loss is around 0.0045 wt%/month.

## Discussion

There is a nearly linear accumulation of CH_4 _in the sample bottles, Figure 3a-b. In all measurements, the concentration of CH_4 _in the controls are lower than in the sample bottles, indicating that CH_4 _was either formed or released from the olivine sand continuously over time at all temperatures. The controls are subtracted from the sample values and a net accumulation is shown in Figure 3. There seems to be CH_4 _accumulation in the buffered experiments even in the bottle without olivine and thus no access to any obvious catalytic sites. These results are most likely due to hydrocarbon release from the rubber septa used for the experiments but also some formation of CH_4 _from the carbon background in the solution.

There is the possibility that CH_4 _is not formed but is released from the olivine crystal structure or from small fluid inclusions or that it could be the result of decomposition of longer hydrocarbons [30]. Microscopic analyses of thin sections do not reveal any gas inclusions and the amount of available surface carbon is not enough to form CH_4 _to the extent that we have recorded.

When calculating the amount of available carbon on the exposed olivine surface using the surface area from the B.E.T. measurements together with the available XPS data of the amount of hydrocarbons, it was found that the amount of hydrocarbons at the total exposed olivine surface is 0.06 nmol, which is much less than the amount of CH_4 _accumulated in the bottles, Figure 3. Altogether, the total amount of available organic carbon species in the blank controls are identified to be about one order of magnitude less than the measured concentrations of CH_4 _in the sample bottles. XPS analyses showed a total carbon content of 7.24 at% in which 5.15 at% are hydrocarbons and 2.09 at% are other types of carbon like-COOR groups (esters) for example, but not carbonates. In Figure 3, showing the amount of CH_4_, the error is larger in the non-buffered bottles possibly because of a larger system response due to the non-regulated pH. Olivine weathering reaction rates are suggested to decrease with increasing pH [34]. That would mean that the weathering rates in this study would slightly decrease in our non-buffered experiments with time in contrast to the buffered samples due to the larger pH increase in the water experiments, which is vaguely indicated in Table 2. However, as mentioned earlier, the ANOVA tests showed no significant difference between the buffered and non-buffered groups. Accumulation rates shown in Table 2 do not show any major differences between the groups, but clear temperature dependence. Sabatier or FTT reactions are the most widely invoked explanations if abiotic CH_4 _is detected in olivine chemical weathering experiments (surface-liquid reactions) [17,19]. FTT reactions are supported by catalysts such as native metals or oxides of Fe, Ni or Cr [23], which are common constituents of natural olivines [35]. XRD and microscopy analyses of the olivine used in these experiments revealed the presence of magnetite (Fe_3_O_4_) and chromite (FeCr_2_O_4_) (Figures 1a and 1b), both of which are known to have a catalytic effect on the FTT reaction [23,36-38]. Hence, catalysts necessary for FTT reactions were clearly present. Also, other specific areas on the olivine grains were observed by SEM to contain potential catalysts, such as an O-free phase with a Fe content of 95.01 atomic% (Figure 1c) and other phases containing Cu, Fe and Ni (Figure 1d). Sabatier or FTT reactions reduce CO_2 _to CH_4 _but are thought to require high temperatures or strong catalysts [1]. The occurrence of efficient catalysts such as magnetite and chromite in our system may explain reactions involving the reduction of CO_2 _into CH_4 _at lower temperatures than expected. Altogether we confirmed the presence of all the components involved in FTT reactions (i.e. H_2_, CO_2_, CO, CH_4_, H_2_O and necessary catalysts), as well as the accumulation of CH_4 _over time at statistically significant rates even at very low temperatures. In order to lower the redox potential enough to reduce CO_2_, it is necessary to have enough H_2 _in the system as well as a good catalyst. The accumulated H_2 _(Figure 4) may both be released from the dissolving minerals themselves [20] and formed through the splitting and reduction of water through the oxidation of solid, ferrous iron [17,19,39]. Standard thermodynamic calculations of the possibility of H_2 _formation using the temperature and pH ranges of our experiments, 30-70°C and pH 7-10 indicate that the ΔG of formation of H_2 _is negative regardless of the conditions within these ranges [40]. At 30°C, the ΔG of H_2 _formation is between -100 to -200 kJ/mol while at 70°C the ΔG is between -150 and -275 kJ/mol indicating that H_2 _is formed even in low temperature reactions through oxidation of Fe^2+^. The calculations are based on the oxidation reaction of Fe^2+ ^to Fe^3+ ^(Eqs. 4 and 5) with the activity set to 1 for all species. If activity was set to values lower than 1, such as the amount of moles of Fe^2+ ^in solution after 1 month of incubation (5.40E-7 mol), the ΔG of H_2 _formation is around 48 kJ/mol and thus not thermodynamic favorable. This means that Fe^2+ ^should be in a solid state in order to reduce water into H_2_. Concentrations used for the calculations are the measured headspace values ranging from 10.6-32.7 ppmv of H_2_. The accumulated concentration of H_2 _after 9 months of incubation is enough to possibly sustain the survival of some strains of methanogenic archaea [41] but the rates of formation are probably too low to sustain any growth. Further studies have to be made to test the possible survival and growth of hydrogenotrophic methanogenic archaea.

Any presence of iron carbides in the samples could contribute to the formation of H_2 _(Eq. 6) but neither ESEM nor XRD analyses revealed any occurrence in the samples and because of this, the formation of H_2 _through carbide oxidation (Eq. 6) is not considered to be important in our experiments.(4)(5)(6)

The accumulation of CH_4 _in the headspace of the incubation bottle could be explained by either the reverse water-gas shift reaction (RWGS, Eq. 7) followed by FTT reaction or oxidation of Fe^2+ ^in the olivine structure with HCO^3- ^in solution (Eq. 8) and the followed FTT reaction (Eq. 3). The RWGS reaction is the formation of H_2_O and CO from the reaction between H_2 _and CO_2_. At low temperatures, the RWGS has to be catalytically driven in order to be thermodynamically favorable [42].(7)(8)

Unless catalytic sites are available on the olivine surface, the RWGS reaction is not thermodynamically favorable at the experimental temperatures. However, reaction 9 is thermodynamically favorable (ΔG is in the range of -97.27 to -248.50 kJ mol^-1^). The formation of H^+ ^in the reaction (Eq. 8) will not lower the pH of the solution due to the buffering effect of silica [43]. With the existence of CO, CO_2 _and H_2_, the formation of abiotic CH_4 _(Eqs. 2 and 3) is thermodynamically favorable at our experimental temperatures (ΔG is in the range of -33.67 to -96.35 kJ mol^-1^).

Random sample bottles from all sample groups, i.e., all temperatures and solution types were picked and measured for dissolved Si and plotted against CH_4 _(Figure 6). Accumulation of CH_4 _in the headspace of the incubation bottles seems to be strongly correlated with Si release into the liquid phase. It is unclear as to why the regression line does not cross nearer the origo. The lag before the Si concentration increase could be due a threshold effect.

Our data suggest a close link between olivine dissolution, serpentinization reactions and CH_4 _formation. This indicates that the changes in CH_4 _concentration, which are easily measured, can be used as a proxy for olivine dissolution in systems similar to ours. If so, the linear accumulation of CH_4 _over time also indicates linear olivine dissolution over the 9-month timescale of our experiments.

The Mg, Ni and Fe ions in solution are showing clear time dependence (Figure 5a, b, c). Below 70°C, there is an increase in elemental concentration in solution but at 70°C there is a strong decrease in dissolved ions, probably due to secondary mineral precipitation, chelation or flocculation. These elements are probably incorporated into new, hydrated minerals such as serpentine or other solid phases at the surface or in solution. Another possibility is that weathering of the natural olivine releases colloids as well as ions in solution, leading to flocculation instead of precipitation. This process does probably not account for the total decrease of elemental concentration seen in the charts, but may be a process in addition to precipitation. Chelation processes, in which organic molecules surround a metal ion and therefore makes it colloidal may also be a possible process, even though the low concentrations of hydrocarbons in the system should keep this process minimal.

## Implications and conclusions

This study illustrates the interactions between water and natural olivine that result in formation of H_2 _and CH_4_. The formation of CH_4 _was observed to be strongly correlated with olivine dissolution rates at temperatures ranging from 30 to 70°C. This may have important implications regarding questions about early life on Earth because high quality electron donors (H_2 _and CH_4_) can be released when water interacts with very common minerals at temperatures suitable for living cells and not just at temperatures above 100°C as previously reported. This substantially expands the range of environments suitable for chemosynthetic organisms on the early Earth. The relationship between the release of Si and the formation of CH_4 _in the headspace of the incubation bottle is linear at low temperatures and at pH values of 8-9, which could be an indicator for the dissolution of olivine. Magnesium and iron release over time is more difficult to monitor due to precipitation as secondary minerals, flocculation or chelation, and may therefore not be used as an indicator for CH_4 _production or olivine dissolution.

## Competing interests

The authors declare that they have no competing interests.

## Authors' contributions

AN drafted the manuscript. AN and ND carried out the experiments. AN, ND, DB and NGH designed the experiment, contributed to the interpretation of the results and discussion. PC participated in PP1 and GC analyses. All authors read and approved the final manuscript.
